# Supplementary material for: HIVprotI: an integrated web based platform for prediction and design of HIV proteins inhibitors
Source: J Cheminform. 2018 Mar 9;10:12. doi: 10.1186/s13321-018-0266-y (PMC5845081; doi:10.1186/s13321-018-0266-y)
Supplement: Supplementary file 2 — Additional file 2. Source code of HIVProtI web server. [file 13321_2018_266_MOESM2_ESM.zip › HIVprotI_Source-code/hivprotI/weka-3-6-6/doc/weka/clusterers/forOPTICSAndDBScan/OPTICS_GUI/package-tree.html]

weka.clusterers.forOPTICSAndDBScan.OPTICS\_GUI Class Hierarchy


---


|  |  |  |
| --- | --- | --- |
| **Overview** \| **Package** \| Class \| **Tree** \| **Deprecated** \| **Index** \| **Help** \| **Weka's home** | |  |
| **PREV**   **NEXT** | **FRAMES**    **NO FRAMES**     **All Classes** |  |


---


## Hierarchy For Package weka.clusterers.forOPTICSAndDBScan.OPTICS\_GUI

**Package Hierarchies:**: All Packages

---

## Class Hierarchy

- java.lang.Object
  - javax.swing.table.AbstractTableModel (implements java.io.Serializable, javax.swing.table.TableModel)
    - weka.clusterers.forOPTICSAndDBScan.OPTICS\_GUI.**ResultVectorTableModel** (implements weka.core.RevisionHandler)
    - java.awt.Component (implements java.awt.image.ImageObserver, java.awt.MenuContainer, java.io.Serializable)
      - java.awt.Container
        - javax.swing.JComponent (implements java.io.Serializable)
          - weka.clusterers.forOPTICSAndDBScan.OPTICS\_GUI.**GraphPanel** (implements weka.core.RevisionHandler)
      - javax.swing.filechooser.FileFilter
        - weka.clusterers.forOPTICSAndDBScan.OPTICS\_GUI.**SERFileFilter** (implements weka.core.RevisionHandler)
        - weka.clusterers.forOPTICSAndDBScan.OPTICS\_GUI.**OPTICS\_Visualizer** (implements weka.core.RevisionHandler)- weka.clusterers.forOPTICSAndDBScan.OPTICS\_GUI.**SERObject** (implements weka.core.RevisionHandler, java.io.Serializable)

---


|  |  |  |
| --- | --- | --- |
| **Overview** \| **Package** \| Class \| **Tree** \| **Deprecated** \| **Index** \| **Help** \| **Weka's home** | |  |
| **PREV**   **NEXT** | **FRAMES**    **NO FRAMES**     **All Classes** |  |


---
